# Supplementary material for: Complexity and 1/f slope jointly reflect brain states
Source: Sci Rep. 2023 Dec 7;13:21700. doi: 10.1038/s41598-023-47316-0 (PMC10709649; doi:10.1038/s41598-023-47316-0)
Supplement: Supplementary file 1 — Supplementary Information. [file 41598_2023_47316_MOESM1_ESM.pdf]

Supplementary Material
Complexity and 1/f slope jointly reflect brain states

Vicente Medel*[a], Martín Irani*[b], Nicolás Crossley[c], Tomás Ossandón[c, d], and Gonzalo Boncompte[c, e]

[a]Latin American Health Brain Institute (BrainLat), Universidad Adolfo Ibáñez, Santiago, Chile
[b]Department of Psychology, University of Illinois Urbana-Champaign, IL, United States
[c]Departamento de Psiquiatría, Escuela de Medicina, Pontificia Universidad Católica de Chile, Santiago, Chile
[d]Institute for Biological and Medical Engineering, Schools of Engineering, Medicine and Biological Sciences, Pontificia Universidad Católica de Chile, Santiago, Chile
[e]División de Anestesiología, Escuela de Medicina, Pontificia Universidad Católica de Chile, Santiago, Chile
[*]These authors contributed equally and share first authorship.

DRAFT

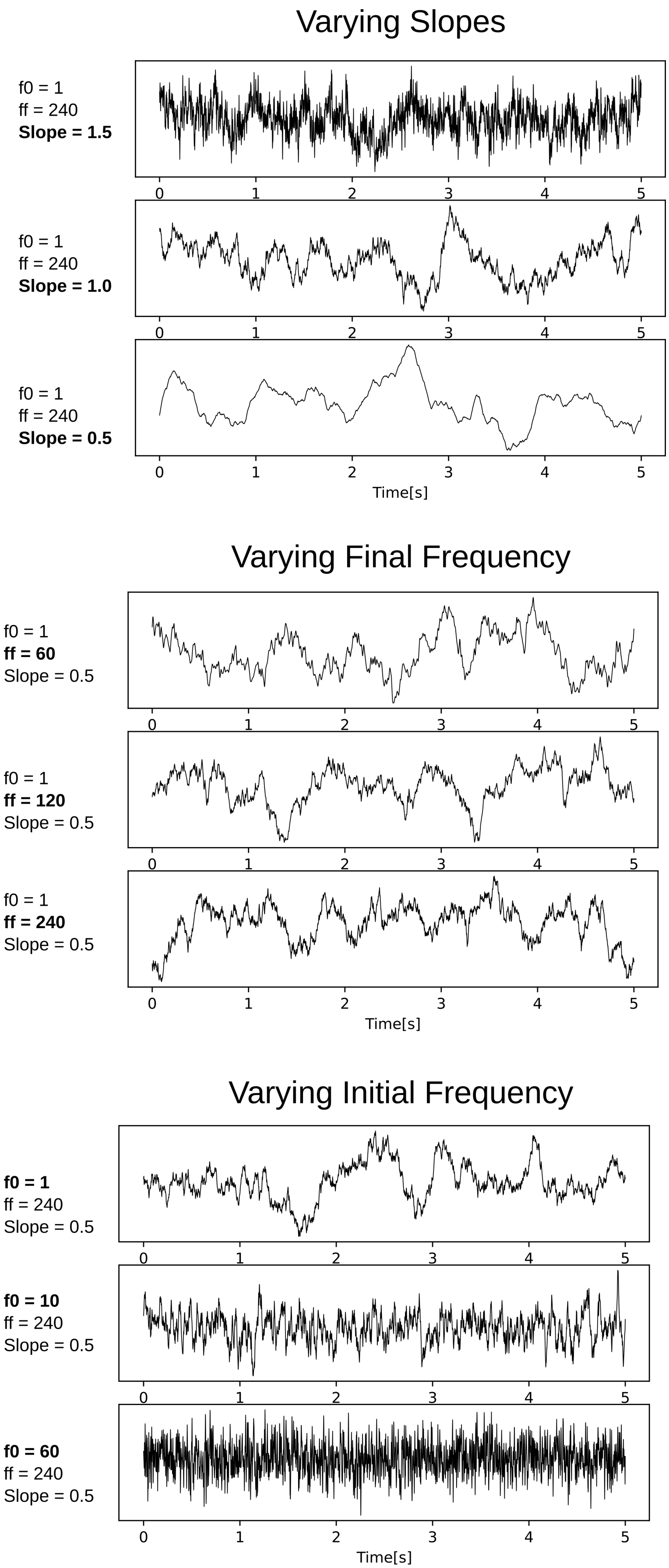

Fig. 1. Supplementary Figure 1. Sample signals with varying iDFT parameters.

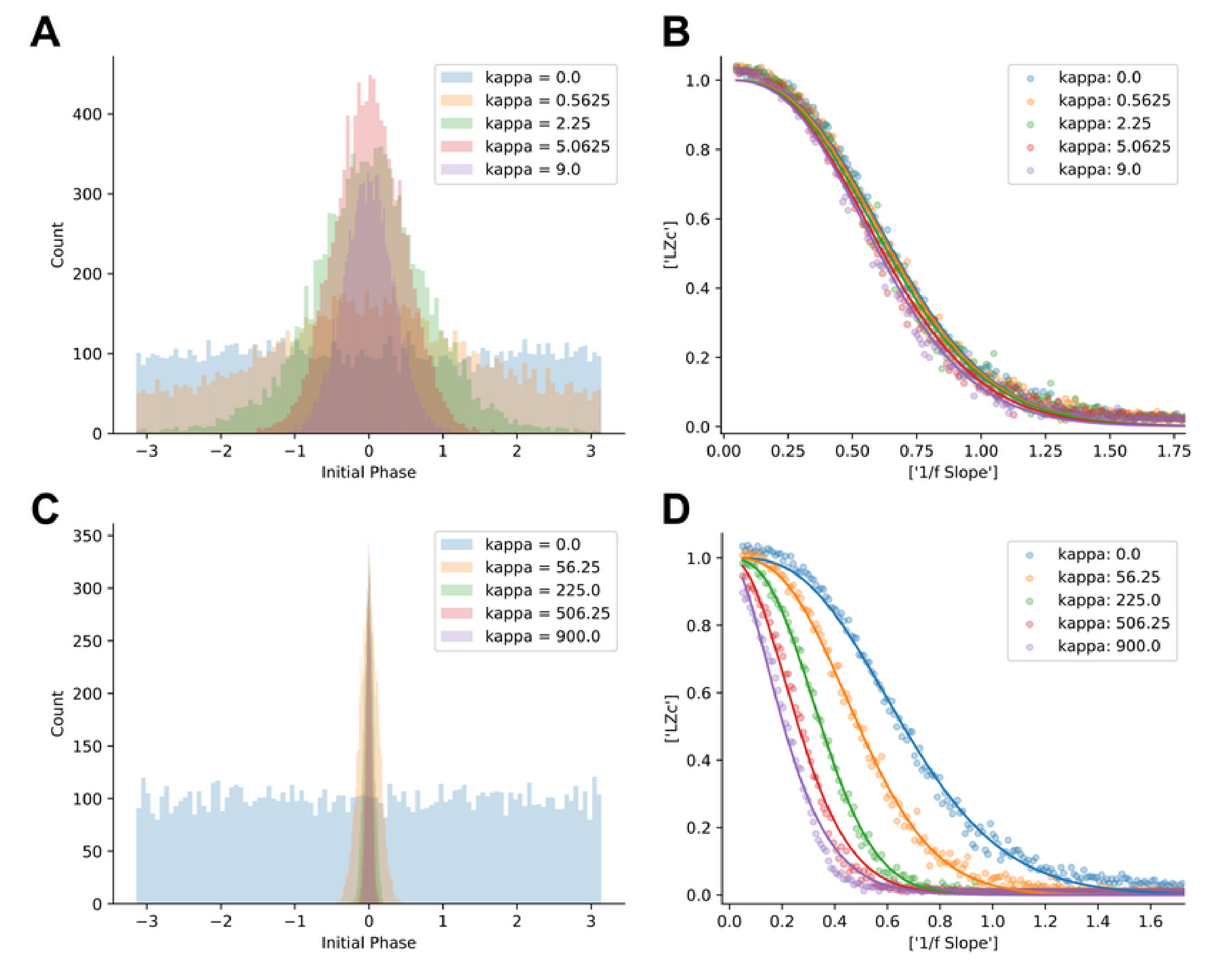

Fig. 2. Supplementary Figure 1. The relation between LZc and 1/f slope in iDFT models generated with different distributions of initial phases. (**A**) Histograms of initial phases of all frequencies used in iDFT models. They follow von Misses distributions with various kappa values. A kappa value equal to 0 generates a distribution of phases that is indistinguishable from an uniform distribution. (**B**) Scatter plot showing the relation between LZc AND 1/f slope for 256 iDFT simulations with varying 1/f slope for the 5 different kappa values shown in A. **C** and **D** are analogous to **A** and **B**, but using much higher kappa values. It can be seen that for physiologically relevant kappa values, LZc is unaffected by the different distributions of initial phases. It is only at very high values of kappa in the von Misses dstribution of initial phases, which impy that all frequencies have essentially the same initial phase, that LZc starts to be significantly reduced.

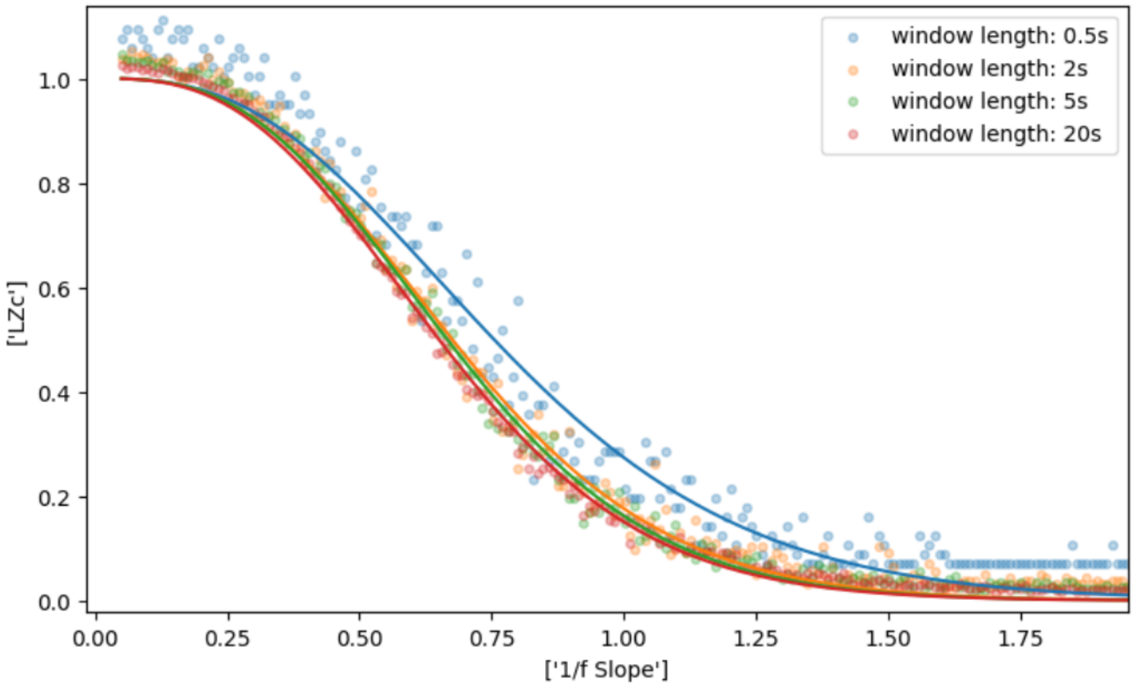

Fig. 3. Supplementary Figure 2. The relation between 1/f slope and LZc is not affected by the window length. Using different window length (0.5s, 2s, 5s and 20s), the relation between 1/f slope and LZc remains similar over 5s window length.

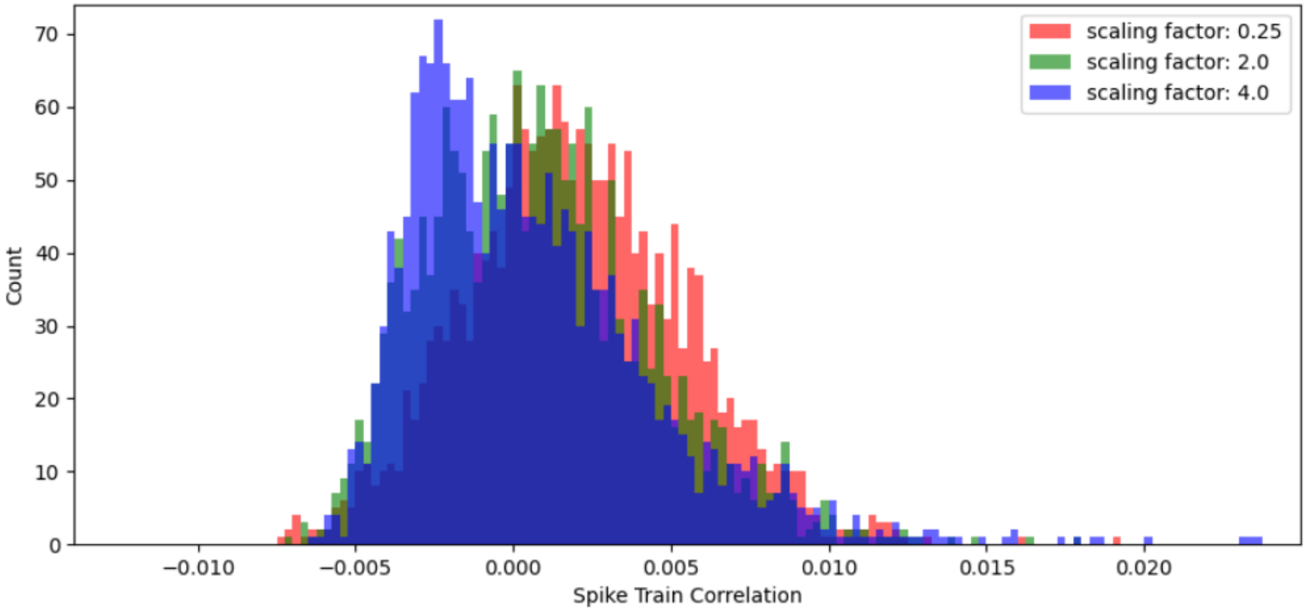

Fig. 4. Pairwise correlation of the LIF model across E/I scaling factors. Simulated 200 neurons across different E/I scaling factors using spike time tiling coefficent histograms in three representative scaling factors show low pairwise correlation values for all regimes, suggesting that all simulations sit within a related asynchronous irregular state.

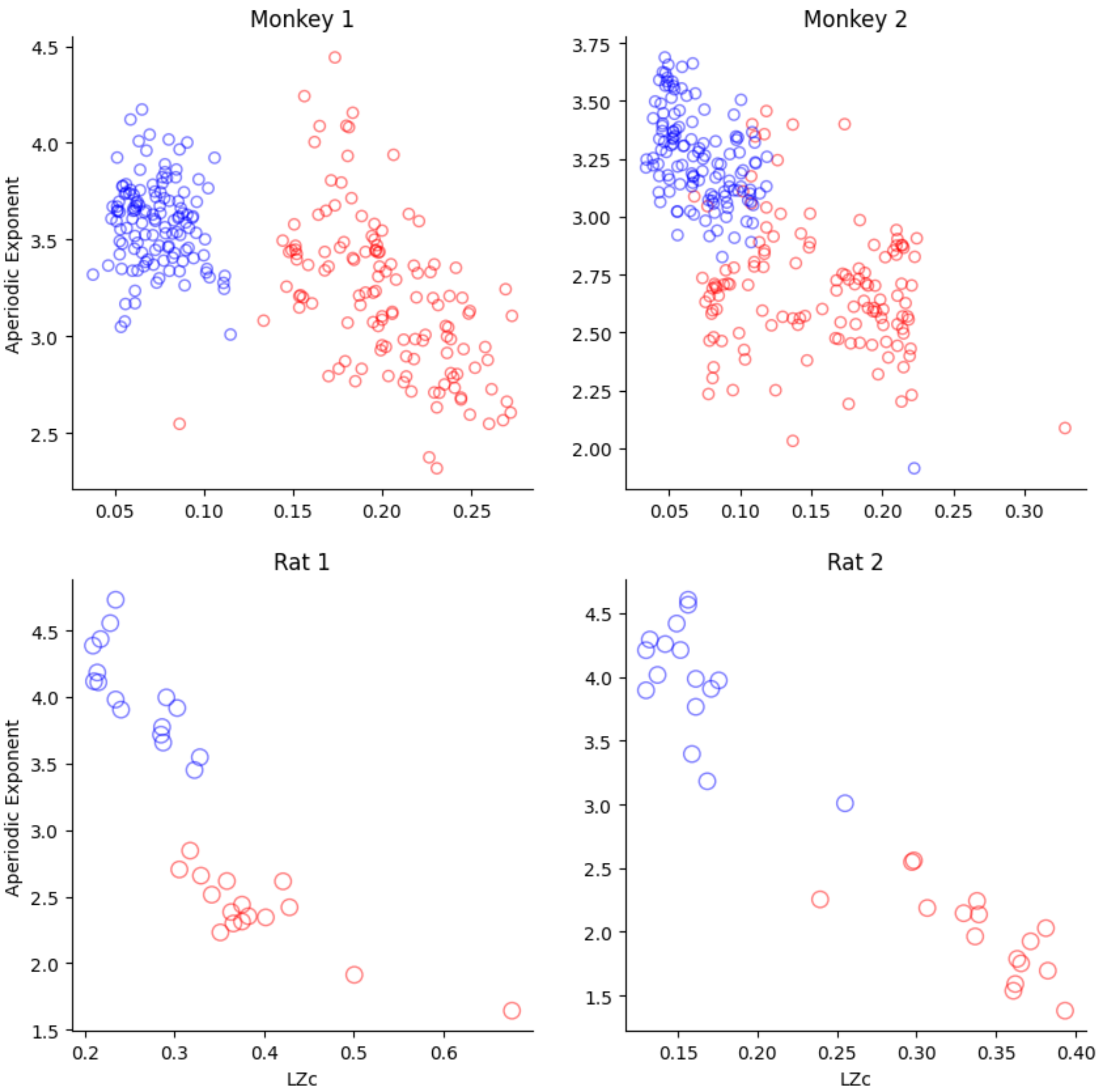

Fig. 5. Spatial correlation structure between 1/f slope and LZc across species and states. In Red, electrodes in Awake, and Blue represents propofol sedation. It can be observed that the correlation structure between the measures maintains across states, delineating the relation proposed in Figure 1 and Figure 2. Interestingly, this relation is stronger in awake state, as compared to propofol sedation (Monkey 1, Awake rho=-0.57 p=1.02e-12, Propofol rho=-0.09, p=0.27; Monkey 2, Awake rho=-0.13 p=0.14, Propofol rho=-0.47, p=1.63e-08; Rat 1, Awake rho=-0.65 p=0.005, Propofol rho=-0.75 p=0.0006; Rat 2, Awake rho=-0.8 p=0.0001, Propofol rho=-0.51 p=0.04).

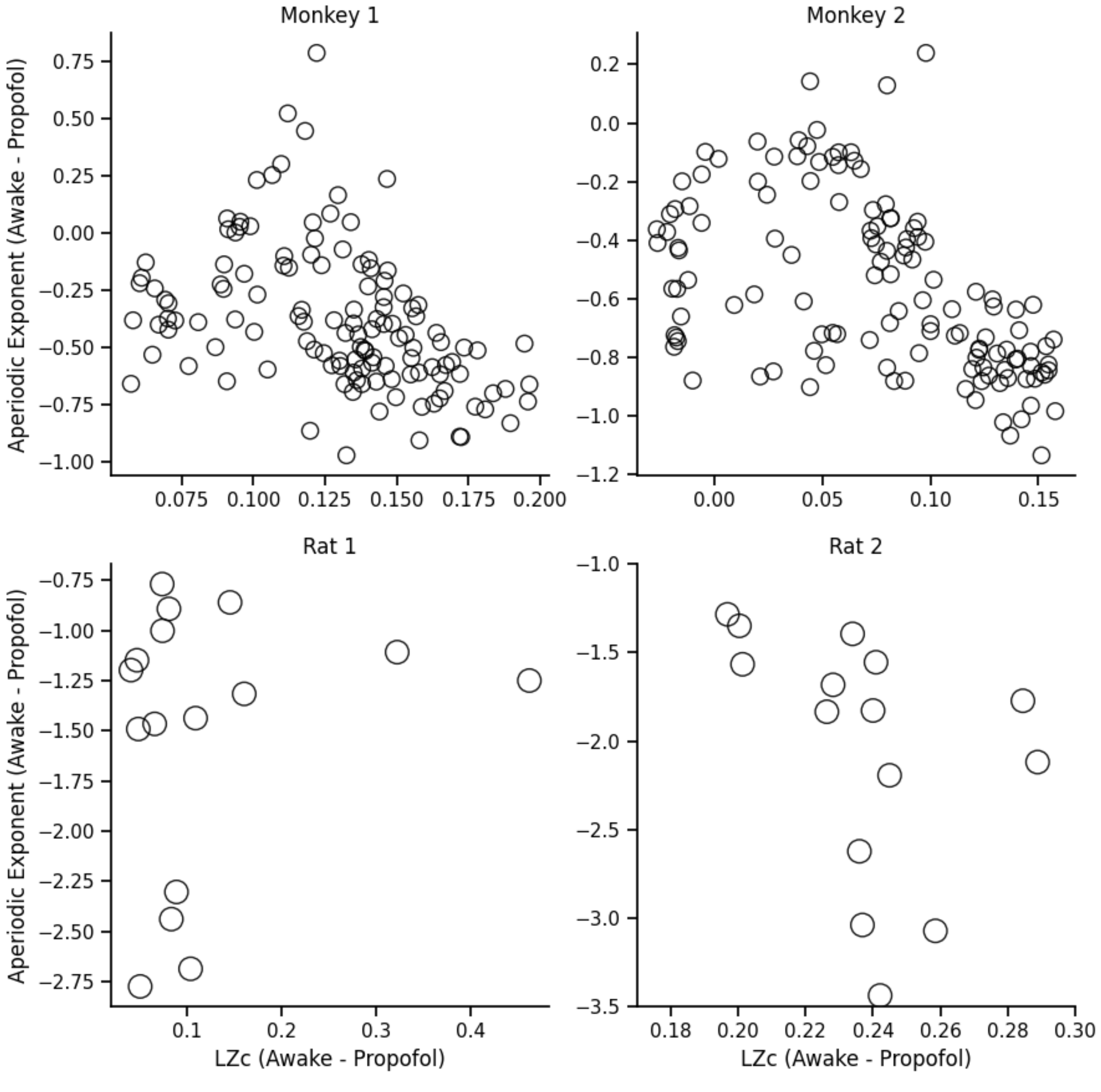

Fig. 6. Spatial correlation structure between delta 1/f slope and LZc across states. Simple linear model, with all animals aggregated and both variables centered (delta LZc and delta 1/f slope) showed a strong inverse relation (adjusted $r^2 = 0.20$, $\beta = -2.96$; $F(1, 286) = 71.04$; $p < 0.001$). Multiple regression model that estimated the linear dependence between these measures for each animal showed a significant main effect (adjusted $r^2 = 0.23$; $F(4, 283) = 21.84$; $p < 0.001$), and significant individual effects for Monkey 1 ($\beta = -3.37$; $p < 0.001$), Monkey 2 ($\beta = -2.641$; $p < 0.001$) and Rat 2 ($\beta = -6.27$, $p < 0.001$), while for Rat 1 the linear dependence did not reach significance ($p = 0.36$).

Manipulated Variable	Frequency [Hz]	Equation Fitted	Best Fitting Value						Goodness of Fit
			a	*std(a)*	*b*	*std(b)*	*c*	*std(c)*	R^2
f_0	1	4	0.030	0.102	4.356	2.852	1.621	0.810	0.997
f_0	10	4	0.130	0.169	2.349	1.452	1.511	0.867	0.997
f_0	30	4	0.277	0.322	1.518	1.378	1.504	1.207	0.996
f_0	60	4	0.441	0.518	1.064	1.630	1.581	1.934	0.992
f_f	30	3	0.214	0.293	2.440	4.209	0.896	2.502	0.981
f_f	60	3	0.347	0.255	2.768	2.663	1.052	1.167	0.989
f_f	120	3	0.564	0.245	3.301	1.979	1.151	1.115	0.994
f_f	240	3	0.812	0.228	3.657	1.635	1.277	0.849	0.995

Eq. number	
**3**	y = a * exp(-b * (ln(x**c + 1))**2)
**4**	y = a + (1 - a) * exp(-b * (ln(x**c + 1))**2)

where x = slope and y = LZc

Fig. 7. Supplementary Table 1. Goodness of fit of iDFT equation 3 and 4.
